# A GC-MS Database of Nitrogen-Rich Volatile Compounds

**DOI:** 10.3390/toxics13110986

**Published:** 2025-11-16

**Authors:** Anastasia Yu. Sholokhova, Svetlana A. Borovikova, Dmitry S. Kosyakov, Dmitriy D. Matyushin

**Affiliations:** 1 Frumkin Institute of Physical Chemistry and Electrochemistry, Russian Academy of Sciences, 31-4, Leninsky Prospect, 119071 Moscow, Russiadm.matiushin@mail.ru (D.D.M.); 2 Laboratory of Environmental Analytical Chemistry, Northern (Arctic) Federal University, 163002 Arkhangelsk, Russia

**Keywords:** gas chromatography, mass spectrometry, electron ionization, retention index, unsymmetrical dimethylhydrazine

## Abstract

Unsymmetrical dimethylhydrazine (UDMH) was previously used as a rocket propellant in launch vehicles. During the operation and accidents of launch vehicles, hundreds of tons of UDMH were released. While these launch vehicles are gradually being phased out, UDMH continues to be used in space technology and other industries. When released into the environment, UDMH forms numerous transformation products. Several dozen have been reliably identified, and hundreds are believed to exist, many of which are highly toxic and quite persistent in the environment. Gas chromatography–mass spectrometry (GC-MS) is one of the primary methods for identifying these compounds. Library searches using mass spectra and retention indices are often used. However, UDMH transformation products are highly specific—they are organic compounds, typically aromatic heterocycles, with unusually high nitrogen content. Such compounds are poorly represented in GC-MS databases, while existing data are often of poor quality and were obtained back in the 1980s. A database of such compounds was presented, containing information on retention indices for non-polar (5%-phenylpolydimethylsiloxane) and polar (polyethylene glycol) stationary phases, as well as electron ionization mass spectra (70 eV) for 104 nitrogen-containing compounds: derivatives of triazoles, pyrazoles, imidazoles, pyridines, diazines, and triazines, as well as amides and other compounds. Many of the compounds presented in the database are proven UDMH transformation products, while many of the other compounds are probable. Derivatives of triazoles and triazines are also used as pesticides, and our database can be useful in detecting their derivatives. The database is free and available online.

## 1. Introduction

Unsymmetrical dimethylhydrazine (UDMH) has been used as a rocket propellant in launch vehicles and missiles worldwide for many decades [1]. UDMH was typically used in combination with nitrogen dioxide as an oxidizer: both compounds are storable as liquids at room temperature, and they are hypergolic. The use of UDMH as a fuel has now declined significantly, with space agencies around the world phasing it out in favor of solid propellants and cryogenic rocket engines [2]. In the past, significant amounts of UDMH were released into the environment during space operations, and the negative impacts of these spills on the environment continue to this day, due to the fact that UDMH forms a variety of persistent and hostile transformation products when exposed to water and air [3,4,5,6]. Smaller amounts of UDMH continue to be released into the environment today [7].

When spent stages of launch vehicles fall, up to several tons of UDMH are released into the environment; in launch vehicle accidents, the amount of UDMH released is measured in tens of tons and causes severe damage to the environment [8]. For example, despite the fact that the fall of the Dnepr rocket at the Baikonur Cosmodrome occurred on 27 July 2006, self-restoration of the soil and vegetation cover was at an early stage in 2024 [9]. Natural restoration of ecosystems after such accidents takes a long time [10], and studies show that the natural soil cover is not fully restored in 10 years or more. The Proton-M launch vehicle accident (2 July 2013) resulted in the rocket falling in the area of the Baikonur Cosmodrome, 2.5 km away from the launch pad [10]. At the time of the fall, the rocket’s fuel tanks contained more than 600 tons of fuel and oxidizer [10]. Upon impact with the ground, the rocket exploded, forming a crater up to 5 m deep and up to 40 m in diameter.

UDMH is an extremely reactive substance that, upon contact with atmospheric oxygen and other oxidizers, as well as during uncontrolled storage of UDMH-containing mixtures, forms hundreds of unusually structurally diverse transformation products [4,6]. Most of the established structures are heterocyclic compounds containing from one to seven nitrogen atoms in their structure [6,11]. However, when monitoring the environment, researchers are usually limited to determining several major components [12,13]. For other compounds, their concentration in environmental objects and their properties often remain unknown. A number of studies have shown that the structures of most of the resulting UDMH transformation products have most likely not yet been established [6]. Thus, the identification of UDMH transformation products in various environmental samples is a very important task.

Gas chromatography–mass spectrometry (GC-MS) is one of the most widely used methods for identifying UDMH transformation products [4,6,11,14]. In this case, rapid screening is usually carried out using the NIST database [4,6,11]. The database contains mass spectra and retention indices (RI). However, it was previously shown that this database contains many incorrect entries, specifically for nitrogen-containing compounds—typical UDMH transformation products and their analogs [15,16]. Thus, with a high degree of reliability for five nitrogen-containing heterocyclic compounds (1,2,4-triazole, 3-amino-1,2,4-triazole, imidazole, 3-methylpyrazole, 3,5-dimethylpyrazole), it was shown that the NIST database contains incorrect RI values. For example, for such simple and well-studied compounds as 1,2,4-triazole and imidazole, the difference between the RI values in the NIST database and the correct ones is more than 100 units [16].

There are various reasons [16] for this: in many cases, the data in the database are taken from quite old publications, and were obtained in the 1980s–1990 using reference samples of unknown origin, without using a mass spectrometer (mislabeling is very likely). In other cases, the database contains values obtained without using reference samples at all—RI of components identified by a simple library search are given as “reference”. This is also very untrustworthy data [16], because this is a very unreliable way of peak annotation, leading to an incorrect result in 10–20% of cases [17].

Even when the NIST database contains multiple entries for the same compound, all of these entries may be equally incorrect [16] (an error of 100 or more units) because when including new entries, the library authors compare them with existing ones (possibly incorrect) and do not include them in case of significant discrepancies [18]. Also, different entries for a single compound may not be truly independent. The database often contains multiple values from the same source; data may be obtained from different publications, but obtained by the same group of authors using the same sample; entries are often obtained from secondary sources that simply duplicate the primary source [16].

The NIST mass spectral database also has a number of shortcomings: the mass spectra were obtained by a variety of authors, in different years, under different conditions. The database contains mass spectra of varying quality: in some cases, low-intensity peaks are not included, and the “correct” isotopic pattern is not observed. The database does not provide important metadata (how and with what instrument the mass spectrum was obtained), and the mass spectra provided there are not always reproduced in subsequent experiments [19].

In addition to database-based identification, a machine learning-based approach can be used [6]. This involves predicting RI [20,21] and mass spectra [22,23,24] based on the molecular structure and using the predicted mass spectra as a reference [6,23]. Molecular fingerprint prediction based on the observed mass spectrum is also used to select the most suitable structure [25]. In all cases known to the authors, the NIST database is used as a training set for training such models in GC-MS. Errors in the database used to train the corresponding models, as well as the database’s incompleteness, also affect the accuracy of the prediction. Models with a very low mean absolute error “on average” demonstrate poor accuracy for UDMH transformation products and their analogs [15].

Taking into account the importance of the task of GC-MS identification of UDMH transformation products and the imperfect nature of data on such compounds in the currently most popular NIST mass spectral and RI database, it is relevant to create a reference database of electron ionization (EI) mass spectra and RI of small nitrogen-containing compounds with a large mass fraction of nitrogen atoms, including known and potential UDMH transformation products. When creating such a database, the task of identifying errors in existing databases is simultaneously solved.

This work aimed to create such a database, obtaining chromatographic and mass spectrometric data for a number of nitrogen-containing volatile organic compounds and publishing them in the public domain. The database includes RI for two stationary phases obtained under various conditions and EI mass spectra. The obtained data can be used in the future for the environmental monitoring of areas affected by rocket accidents and for identifying UDMH transformation products.

## 2. Materials and Methods

### 2.1. Sample Preparation

The library was compiled using pure samples of compounds purchased from Sigma Aldrich (St. Louis, MO, USA), Shanghai Macklin Biochemical Technology (Shanghai, China), and other vendors, and also those available at the Institute of Physical Chemistry and Electrochemistry and Northern (Arctic) Federal University. All samples were purchased from vendors specializing in research-grade reagents, or their structure was confirmed by reliable methods such as high-resolution mass spectrometry and nuclear magnetic resonance. The declared purity of all samples was at least 98%. Purity was verified by GC-MS and high-performance liquid chromatography; no impurity chromatographic peaks were observed.

To prepare stock solutions of the analytes, 1 mg (1 µL for liquid samples) of each compound was dissolved in 1 mL of HPLC-grade methanol (EVA Science, Saint-Petersburg, Russia) and stored at −20 °C in a laboratory freezer. Solutions of analyte mixtures used to determine the RI were prepared by mixing and then diluting the stock solutions with methanol. Solutions containing 1–10 substances in methanol were then prepared at a concentration of ~0.1 mg/mL each. The concentrations of substances in the analyzed solutions were selected to obtain a sufficiently intense signal to extract a high-quality mass spectrum. These solutions were used for obtaining RI and mass spectra. The solutions were prepared to avoid any overlapping of chromatographic peaks, as well as the inclusion of isomers in a single solution. This allows for unambiguous peak annotation and the extraction of mass spectra from fully resolved peaks.

Individual alkanes from C5 to C14 (1.0 mg/mL of each component in methanol) and a standard set of C7–C40 alkanes (1 mg/mL of each component in hexane, Sigma-Aldrich) were used to determine the RI. Mass spectra for the database were extracted from mass chromatograms of mixtures not containing added *n*-alkanes.

### 2.2. GC-MS Analysis

A Shimadzu GCMS-TQ8040 gas chromatograph with mass spectrometric detection (Shimadzu Corporation, Kyoto, Japan) was used with a Split/Splitless Liner (part number 221-75193; Shimadzu Corporation, Kyoto, Japan). Helium (99.9999%) was used as carrier gas. Two chromatographic columns were used: with a non-polar (5%-phenylpolydimethylsiloxane) and polar (polyethylene glycol) stationary phase, respectively: (i) HP-5MS, 30 m × 0.25 mm × 0.50 μm, Agilent (Santa Clara, CA, USA); (ii) HP-INNOWax, 30 m × 0.25 mm × 0.25 μm, Agilent. The test mixture of *n*-alkanes and the studied substances was separated using gradient modes with various heating rates. The “linear velocity” flow control mode was used. For the non-polar stationary phase, the following conditions were used: the temperature ramping 40–320 °C; heating rate 6, 9, or 12 °C/min; then 10.0 min hold at 320 °C; linear velocity 33.0 cm/sec; injector temperature 250 °C. For the polar stationary phase, the following conditions were used: the temperature ramping 40–240 °C; heating rate 6, 9, or 12 °C/min; then 10.0 min hold at 240 °C; linear velocity 36.3 cm/s; injector temperature 200 °C. Each sample was analyzed three times in all modes. In all cases, 0.5 µL of sample was injected in flow split mode (split 1:20). The split rate was chosen to ensure acceptable intensity and a high-quality mass spectrum, but to avoid overloading the mass detector. Mass detector parameters: ion source temperature 200 °C; interface temperature 250 °C; electron energy: 70 eV; scanning speed: 0.3 s/scan. Electron ionization (MS1) was used, and a full scan with an *m*/*z* range of at least 40 to 500 was recorded. Perfluorotributylamine was used for auto-tuning, and ions with *m*/*z* values of 69, 219, and 502 were considered.

Some of the RI data (32 molecules) included in the database were taken from a previous work by our research group [15]. The mass spectra of these 32 compounds were also extracted from previously obtained data under slightly different conditions, as given in the corresponding work [15].

### 2.3. Data Processing and Database Assembly

Shimadzu LabSolution GCMS Solution (version 4.50) software was used for data extraction. Background subtraction was performed. The resulting mass spectra were saved in text format and then converted to MSP, SDF, and JDX (JCAMP) file formats for use with MS Search software (version 3.0). All mass spectra were searched in the NIST 23 database, and the one with the highest match factor (MF) value was selected from several replicates. Two spectra exhibited very low MF values (less than 800, see below); these cases were manually reviewed; details are provided in the Section 3. For 12 molecules, a corresponding mass spectrum was missing from the NIST 23 database. All spectra for these 12 compounds were manually analyzed (peak by peak) and compared with the predicted mass spectra. If multiple mass spectra for a single compound (molecules not in NIST 23) were of sufficient quality, based on manual review, one of them was arbitrarily selected for the database. The spectra were predicted from structure using the CFM-ID software (version 2.4) [22] and the gcms-id.ca web service [23]. All mass spectra were checked for noise, and the observed *m*/*z* values were consistent with the structure. Peaks with intensities less than 1/999 of the intensity of the most intense peak (base peak) were discarded. Peaks in the high *m*/*z* region (significantly greater than the molecular weight of the analyte) were discarded; such impurity peaks with low intensity were observed in rare cases; *m*/*z* values were rounded to half-integer numbers. A small number of doubly charged fragment ions were observed, so it was chosen not to round to integers. However, (in accordance with the work [26]), *m*/*z* values above 0.62 were rounded up to the nearest whole number. Intensity values were rounded to integers from 1 to 999.

RI values were calculated using *n*-alkane peaks (linear retention indices, temperature programming mode):

$$I=100\;\times \frac{t-t_{n}}{t_{n+1}-t_{n}}+100n$$

where *I*—retention index; *t*, *t_n_*, *t_n_*_+1_—retention times of the analyte and adjacent previous and subsequent alkanes, respectively; *n*—the number of carbon atoms in the preceding alkane.

An *n*-alkane solution (see above) containing *n*-alkanes adjacent to the analyte was added directly to the solution containing the analyte (except in cases of extreme peak overlap between the analyte and *n*-alkane). The value recorded in the database is the average of three replicate experiments (under identical conditions). Three measurements were performed for each of the three heating rates (6, 9, and 12 °C/min). In all cases, the spread between the minimum and maximum of the three measurements under identical conditions did not exceed 5 units, and in most cases, it did not exceed 1.5 units. Some RI data were obtained from a previous study by our group [15]. All mass spectra are published (in machine-readable form) for the first time.

## 3. Results and Discussion

### 3.1. The mini-N_i_ GC-MS Database

**Figure 1:**
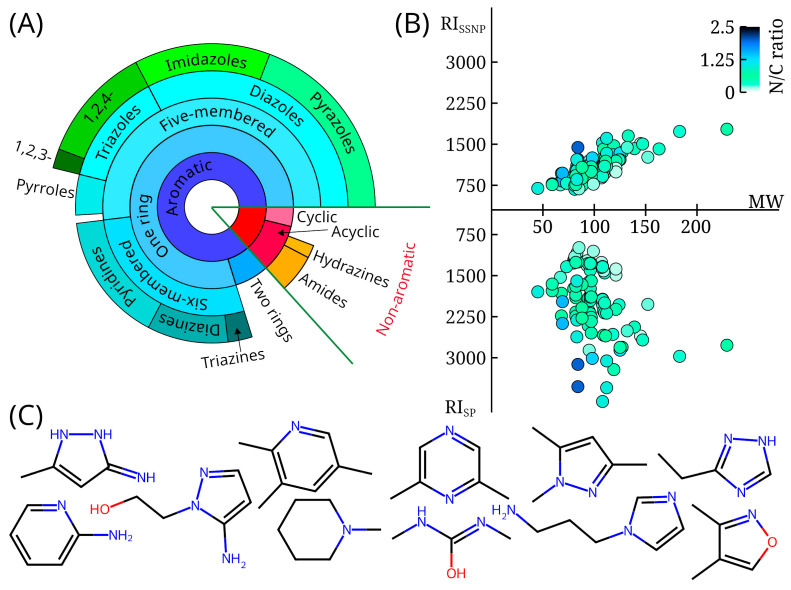
(**A**) The diversity of compounds with mass spectra and retention indices available in the *mini*-N*_i_* database; the distribution of compounds is shown as a tree diagram, with the size of sectors within each concentric ring proportional to the number of molecules; (**B**) distribution of retention indices for non-polar (RI_ssnp_) and polar (RI_sp_) stationary phases, as well as molecular weight (MW) for all molecules available in the *mini*-N*_i_* database; the color of the marker indicates the nitrogen-to-carbon ratio (N/C ratio); (**C**) examples of compounds randomly selected from the *mini*-N*_i_* database (3-amino-5-methylpyrazole, 2,3,5-trimethylpyridine, 2,6-dimethylpyrazine, 1,3,5-trimethyl-1H-pyrazole, 3-ethyl-1H-1,2,4-triazole, 2-aminopyridine, 2-(5-aminopyrazol-1-yl)ethanol, 1-methylpiperidine, 1,3-dimethylurea, 1-(3-aminopropyl)imidazole, dimethylisoxazole).

RI data and EI mass spectra were obtained for 72 molecules. RI information for another 32 molecules had been published previously [15], but the mass spectra are publicly available for the first time. These data form a database that was called *mini*-N*_i_*, where N*_i_* means “*i* nitrogen atoms”. All compounds in the database contain at least 1 nitrogen atom, and 81 compounds contain at least 2 nitrogen atoms. Figure 1 shows the distribution of the compounds in the database by class (90 aromatic, 14 aliphatic), by RI values on polar and non-polar stationary phases, and by molecular weight. The nitrogen-to-carbon ratio and example structures are also shown. More than half of the data set consists of five-membered aromatic heterocycles with one ring. A significant fraction of compounds are triazoles and pyrazoles. These compounds are typical UDMH transformation products [4,6,11,14].

The database contains the files minini.xlsx, minini.msp, minini.jdx, minini.sdf, minini.csv.zip. The minini.xlsx file is a spreadsheet that includes all the data in a compact form. The spreadsheet contains the following columns: “N” (number or id of the compound), “Name”, “SMILES”, “InChI”, “CID” (number in PubChem database), RI values for non-polar (denoted as SSNP) and polar (denoted as SP) stationary phases, “K” (this column shows whether the RI data are taken from [15]), “InChIKey”, RI values for different heating rates (6, 9, 12 °C/min; not available if the RI data are taken from [15]), “MF” (molecular formula), “MW” (molecular weight), “Mass spectrum”. The mass spectrum is given as *m*/*z*-intensity pairs in parentheses, for example, “(59.0, 999)”. The minini.msp, minini.jdx, and minini.sdf files contain mass spectra in MSP, JDX (JCAMP-DX), and SDF formats. Any of these files can be imported into NIST MS Search (minini.msp is recommended), as well as other similar software. The minini.msp and minini.sdf files also contain retention index values. All of the above files contain information about RI, molecular structures (SMILES and InChI strings; InChIKey identifier), names, and mass spectra. The most complete information is given in the minini.xlsx file. The minini.csv.zip file contains mass spectra in CSV (comma-separated values) format; each file contains one mass spectrum. The file names correspond to the database number (column “N” in the minini.xlsx file). The recommended way to use the database is to import the minini.msp file into NIST MS Search and then use the capabilities of this software.

Many compounds present in the data set, such as 1,3,5-trimethyl-1H-pyrazole, (dimethylamino)acetonitrile, 1-ethyl-1H-1,2,4-triazole, formamide, N-methylformamide, 3-methylpyridine, 2,3-dimethylpyrazine, 2,5-dimethylpyrazine, 2,6-dimethylpyrazine, 2,3,5-trimethylpyridine, 1-ethyl-1,2,4-triazole, 1,3-dimethyl-1H-pyrazole-4-carbonitrile, 3-methylpyrazole. 1-methyl-1H-1,2,4-triazole, 1H-1,2,4-triazole, and 1H-1-methylpyrazole were previously mentioned [6,11,13,14] as possible UDMH transformation products. Many other compounds are structural analogs (for example, positional isomers), quite close in structure to the known UDMH transformation products. These compounds are also very likely to be UDMH transformation products, although they have not been described as such in the literature.

**Figure 2:**
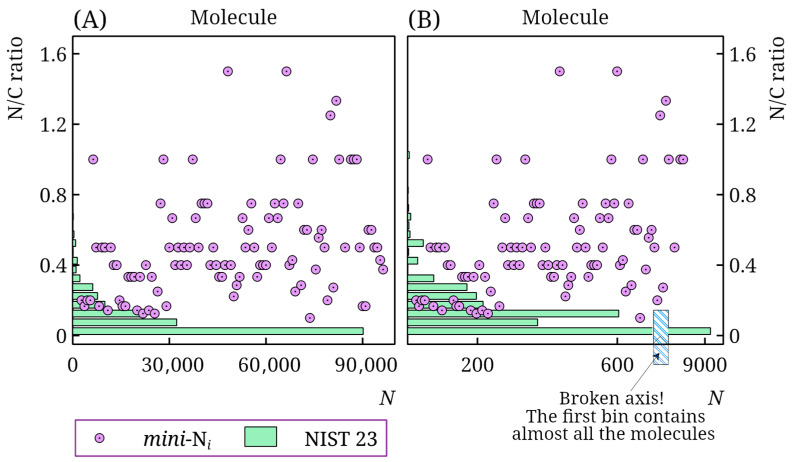
Distribution of molecules in the NIST 23 and *mini*-N*_i_* retention index databases by nitrogen-to-carbon ratio (N/C ratio) for non-polar (**A**) and polar (**B**) stationary phases; for the *mini*-N*_i_* database, the N/C ratio is shown for all molecules (in arbitrary order), for the NIST 23 database, the number of molecules *N* in a bin is shown; note the axis break in subplot (**B**).

Figure 2 shows the distribution of compounds by nitrogen-to-carbon ratio in the NIST 23 database and the *mini*-N*_i_* database. It is evident that, despite the large size of the NIST 23 database, only a small fraction of compounds are nitrogen-containing compounds, in general, and, in particular, nitrogen-rich molecules. This is especially pronounced for the standard polar stationary phase. The NIST 23 database contains RI values for such stationary phases for approximately 11,000 molecules. However, only 57 contain at least 3 nitrogen atoms, and only 33 have a nitrogen-to-carbon ratio above 0.5. Thus, the *mini*-N*_i_* database significantly increases the amount of such data compared to that already available. Other databases [27] also contain virtually no such information.

**Figure 3:**
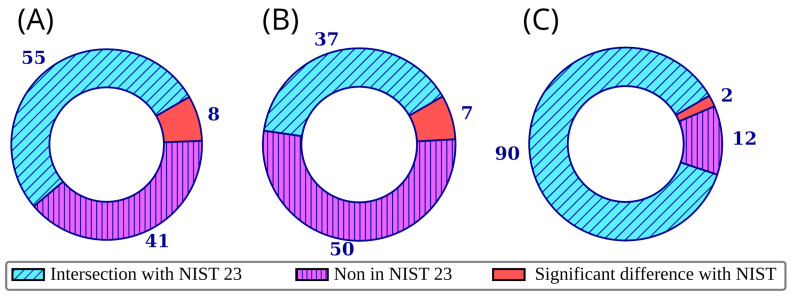
Overlap between the *mini*-N*_i_* database and the NIST 23 database for retention indices on a non-polar stationary phase (**A**), retention indices on a polar stationary phase (**B**), and mass spectra (**C**); each ring corresponds to all compounds in the *mini*-N*_i_* database; significant differences are defined as differences in retention indices of 70 and 100 units (non-polar and polar stationary phase, respectively); the discrepancy between mass spectra in the *mini*-N*_i_* database and NIST 23 is discussed below.

Figure 3 shows the overlap between the NIST 23 and *mini*-N*_i_* databases. The data are presented both for molecules for which information was published for the first time in *mini*-N*_i_* and for the 32 molecules for which the *mini*-N*_i_* RI data were taken from a previous study by our group [15]. For the construction of Figure 3, significant differences are defined as differences in RI of 70 and 100 units (non-polar and polar stationary phase, respectively), or a match factor (Identity algorithm [28,29]) between the library and observed mass spectra less than 800 (see below). The median of all values given for the corresponding types of stationary phase was considered as the RI value from NIST 23. For non-polar stationary phases, the median of all values for the “standard non-polar” and “semi-standard non-polar” stationary phase types was considered. The cases of discrepancies between the NIST 23 and *mini*-N*_i_* data are discussed in more detail in the next section. It should be noted that only the most significant dissimilarities between the databases are shown in Figure 3 and discussed in the next section, while smaller differences also exist.

Table 1 shows the RI data for those compounds for which RI information for at least one of the two stationary phases (polar or non-polar) is missing from the NIST 23 database, but is present in the *mini*-N*_i_* database. Table 1 does not include the data that were taken from the previously published work [15] in the *mini*-N*_i_* database. In total, Table 1 contains 37 compounds for which *mini*-N*_i_* contains data not presented in NIST 23. Of these, in 26 cases NIST 23 lacks information for both types of stationary phases, in 2 cases only for the non-polar stationary phase, and in 9 cases only for the polar stationary phase. There are also 6 more compounds (4-methylpyrazole, 4-methylimidazole, dimethylaminoacetonitrile, 1-methylpyrrolidine, 4-methylpiperidine, 1-methylpiperidine) for which neither NIST 23 nor publication [15] contains retention data for semi-standard non-polar stationary phases (i.e., 5%-phenylpolydimethylsiloxane), but NIST 23 does contain data for polydimethylsiloxane and polyethylene glycol. In all cases except 4-methylimidazole (see below), the RI values observed for 5%-phenylpolydimethylsiloxane are close to those given in NIST 23 for polydimethylsiloxane. These molecules were not included in Table 1 (all data are presented in the published database).

**Figure 4:**
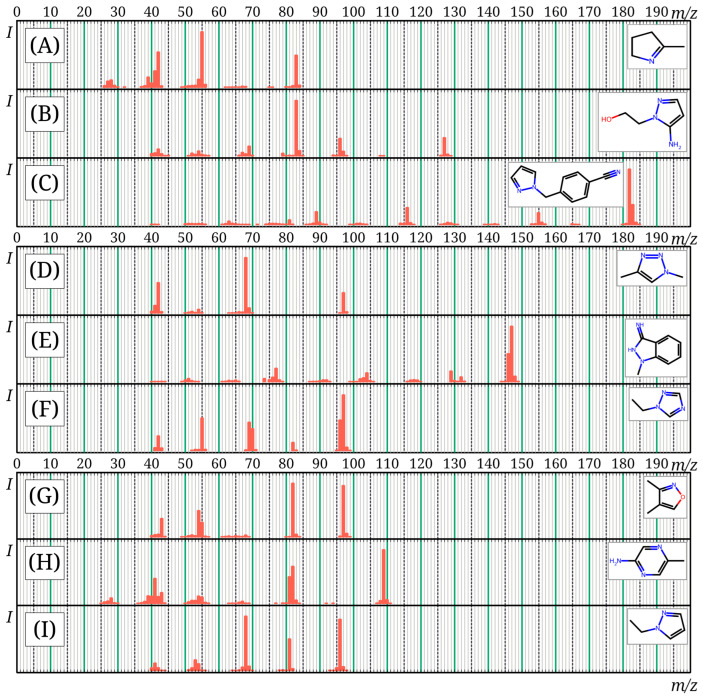
Mass spectra of compounds from the *mini*-N*_i_* database, not included in the NIST 23 database, not previously published: 2-methyl-1-pyrroline (**A**); 2-(5-aminopyrazol-1-yl)ethanol (**B**); 4-(1H-pyrazol-1-ylmethyl)benzonitrile (**C**); 1,4-dimethyl-1H-1,2,3-triazole (**D**); 1-methyl-1H-indazol-3-amine (**E**); 1-ethyl-1H-1,2,4-triazole (**F**); dimethylisoxazole (**G**); 2-amino-5-methylpyrazine (**H**); 1-ethylpyrazole (**I**).

The mass spectra of 12 compounds presented in the *mini*-N*_i_* database are not available in the NIST 23 database. These mass spectra (except for three molecules discussed earlier [15]) are presented in Figure 4; machine-readable versions are given in the *mini*-N*_i_* database. Ultimately, it can be concluded that the *mini*-N*_i_* database does not duplicate NIST 23 and existing databases, but complements them and can be used for the analysis of nitrogen-rich compounds. The resulting database was published online: https://doi.org/10.6084/m9.figshare.30185032, accessed on 9 November 2025.

### 3.2. Inconsistencies with the NIST Database

By far the largest and most widely used RI database is the NIST 23 database. It is a compilation of data obtained over the years and of various origins. For many compounds, the database contains RI values obtained from several different sources. However, often, the original data are difficult to consider reliable [16]. For many compounds, all RI values were obtained in the 1970s–1990s, without the use of mass spectrometers on equipment significantly different from modern ones. In other cases, the data may be taken from articles published in journals with low bibliographic characteristics or from non-peer-reviewed and non-authoritative sources. Finally, the database may contain RI values obtained without the use of standard samples at all, i.e., from the results of tentative analysis of a complex mixture using library searching. All such values are unreliable [16].

Our previous studies [15,16] are devoted to a detailed examination of errors in this database. In particular, it was shown that many values for nitrogen-containing compounds are erroneous (up to 10% of all values) [15]. It was also shown that the presence of multiple entries in the database does not guarantee their accuracy. Entries are not independent; authors of subsequent studies use previously published RI for identification, and an incorrect earlier value can lead to misidentification and the reproduction of errors [16]. When creating a database, the creators include new values for the same molecule only if they do not contradict previous ones [16,18]. This results in the correct value not being accepted due to errors previously included in the database.

In our previous work [16], for some of the most important cases (e.g., imidazole), it was demonstrated beyond a reasonable doubt that the NIST database values were erroneous. Various chromatographic columns from different manufacturers, a variety of chromatographic modes, and confirmation of the structures of all samples using nuclear magnetic resonance were used, as well as repetition of the experiments several times independently. It is important to note that this was not the goal of this study for each molecule. Below, cases where differences between *mini*-N*_i_* and NIST data were observed are listed. This may be due to the fact that the NIST data are erroneous. However, it is not claimed to have definitively established that this explanation is correct.

Table 2 contains molecules (except three cases discussed in a previous work [16]) for which there is a difference of more than 70 units between our RI (mean of 3 values, heating rate 6 °C/min) and the median RI (non-polar stationary phases) from the NIST 23 database. In the case of 1H-indazole, the median value for all non-polar stationary phases is 1321, which is close to the observed value in this work (hence, 1H-indazole is not counted in Figure 3A), but each of the values is significantly different. The difference between the minimum and maximum values obtained in all experiments (3 heating rates: 6, 9, 12 °C/min, 3 repeated experiments for each) is also shown in Table 2. It can be seen that the difference between the *mini*-N*_i_* and NIST values is quite large and cannot be explained by differences in temperature programs.

In the case of 4-methylimidazole, the NIST 23 database lists two identical values. However, both values were measured by the same team and were most likely obtained using the same reference material, so these values do not independently confirm each other. Previously, using reliable reference materials whose structures have been confirmed by nuclear magnetic resonance, it was demonstrated that another work of the same team contains incorrect RI values for nitrogen-containing compounds [16]. This does not mean that this value is also erroneous, but it cannot be ruled out. The NIST database is a secondary source, aggregating data from publications of varying quality.

The NIST 23 database also lists two consistent values given from different sources for 2,4-dimethylpyrrole. However, the newer source [30] used the value from the NIST database to identify 2,4-dimethylpyrrole in a complex mixture, without using reference standards. Therefore, it is possible that the authors of the newer source were misled by the value from the older source via the NIST database if it is assumed that the older source is indeed wrong.

For 1H-indazole, the NIST 23 database lists two values (for the standard non-polar and semi-standard non-polar stationary phases) that differ by 123 units. Such a difference between similar stationary phase types is not observed in any other case, indicating that at least one of the values is erroneous.

For the remaining three molecules, the NIST 23 database contains only one value for non-polar stationary phases. In two cases (2-aminopyrimidine and 2,6-diaminopyridine), the values were obtained by researchers whose values had previously been found to be erroneous. In the remaining case (N,N-dimethylethylenediamine), the RI was obtained by a highly unreliable method, by identifying volatiles using a library search (without using reference materials) in squid.

Thus, in all cases listed in Table 2, the data in the NIST 23 database are given from publications that are not fully reliable and have not been independently verified. The NIST 23 database in this case serves as a secondary source; the data were not obtained directly from the NIST mass spectrometry data center. Of course, it cannot be stated with certainty that the values in the NIST 23 database, rather than those in the *mini*-N*_i_* database, are erroneous. However, it can be recommended that researchers use the values from the *mini*-N*_i_* database in the future. Unlike some of the NIST values, all of these were obtained using pure (not mixed) samples and a mass spectrometer, not non-selective detectors.

Table 3 lists the molecules for which a difference in RI (polar stationary phase) of over 100 units is observed between the *mini*-N*_i_* database and the data given in the NIST 23 database, except for two cases previously discussed in [16]. The spread between the various experimental values obtained in this study is somewhat larger than for the non-polar stationary phase. This is due to the more pronounced dependence of the retention index on the heating rate. However, the variability of the retention index with changes in the temperature program is still much smaller than the difference between the values in the *mini*-N*_i_* and NIST databases.

In these cases, the NIST 23 database contains significantly more values, but the number of sources is smaller than the number of values (the database includes several values from a single publication). As can be seen from Table 3, all values are either taken from very old papers or obtained using highly unreliable methods. In the 1970s and 1980s, in the countries where these RI values were obtained, the quality of commercially available samples was lower, and nuclear magnetic resonance and mass spectrometry were less readily available than they are today. The authors primarily used non-selective detectors. Therefore, although the NIST database contains multiple values for each compound (for the polar stationary phase), it cannot be ruled out that they are erroneous. However, the reason for the discrepancy between NIST and *mini*-N*_i_* for the polar stationary phase has not been sufficiently studied and may be a topic for further research.

**Figure 5:**
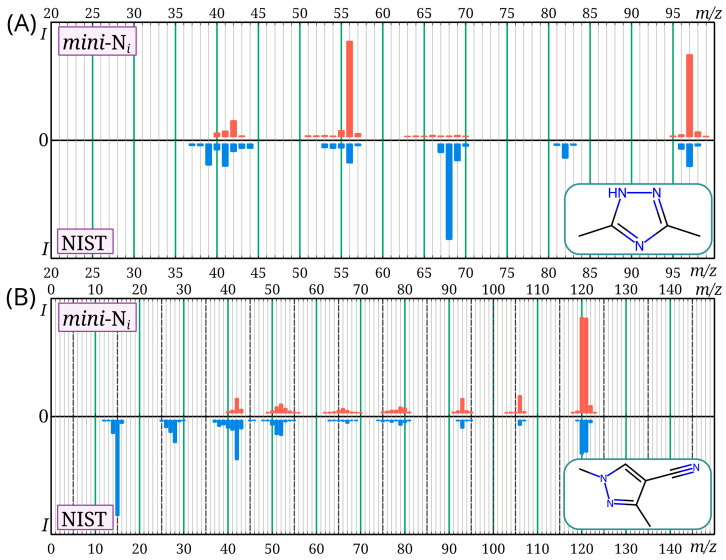
Mass spectra of 3,5-dimethyl-4H-1,2,4-triazole (**A**) and 1,3-dimethyl-1H-pyrazole-4-carbonitrile (**B**) provided in the NIST 23 and *mini*-N*_i_* databases; there is a noticeable discrepancy in the mass spectra for these compounds between the databases.

For the mass spectra, the match factor calculated using the Identity algorithm [28,29] was used as a similarity criterion between our mass spectra and those from the NIST 23 database. In all cases except two, it was at least 840 (out of a possible 1000), which corresponds to satisfactory similarity [31]. For 3,5-dimethyl-4H-1,2,4-triazole and 1,3-dimethyl-1H-pyrazole-4-carbonitrile, the match factor values were 414 and 799, respectively. Thus, in two cases, there is a significant discrepancy between the mass spectra from the NIST 23 database and the *mini*-N*_i_* database. The corresponding pairs of mass spectra are shown in Figure 5. For 3,5-dimethyl-4H-1,2,4-triazole, a large difference between the mass spectra is observed. For all similar compounds (methyltriazoles, methyldiazoles), the molecular ion peak is very intense; in this case, it is not very intense in the mass spectrum from the NIST database. Such a low molecular ion intensity was not observed for any of the similar compounds. In the case of 1,3-dimethyl-1H-pyrazole-4-carbonitrile, the difference between the mass spectra is less significant (this spectrum was recorded with an *m*/*z* range of 40–500). The reasons for the differences between the mass spectra observed in the current work and in the NIST database for these two molecules are unknown.

Thus, it is possible that at least some of the discrepancies between NIST 23 and *mini*-N*_i_* are due to errors in NIST 23, particularly in the RI. The *mini*-N*_i_* database can be used by analytical chemists when analyzing mixtures containing nitrogen-containing heterocycles. In particular, this database is particularly well-suited for identifying UDMH transformation products in various samples, an important task in environmental analysis.

## 4. Conclusions

In this work, a data set was created containing electron ionization mass spectra (70 eV) and linear (temperature programming mode) retention indices for three heating rates for two stationary phases: non-polar (5%-phenylpolydimethylsiloxane) and polar (polyethylene glycol). The data set contains information on 104 molecules.

It was shown that the NIST 23 database (the current main GC-MS database) contains limited data for nitrogen-rich compounds. Particularly limited data are available in the available sources on the retention of nitrogen-rich compounds on polar stationary phases. NIST 23 only provides retention index information for polar stationary phases for only 56 compounds containing at least three nitrogen atoms. However, such chromatographic columns are used in practice for the analysis of such compounds, and such reference data may be in demand. For 35 compounds, the retention index is being published for the first time in this work for a polar stationary phase, and for 28 compounds, the retention index is being published for the first time for a non-polar stationary phase. Thus, it can be concluded that the created data set does not duplicate existing databases, but rather complements them, particularly with regard to retention indices for polar stationary phases.

The database developed in this work contains GC-MS data for a variety of heterocyclic compounds: derivatives of pyrazole, triazoles, imidazole, diazines, pyrrole, pyridine, and others, including amino derivatives. This database can be used to analyze mixtures of UDMH transformation products and identify the corresponding compounds at launch vehicle accident sites, as well as in other applications.

Finally, it should be noted that high-quality data are essential for creating and testing machine learning models. In recent years, impressive progress has been made in predicting retention indices and mass spectra using neural networks. However, for effective model building, similar compounds must be present in the training set. The small number of nitrogen-rich molecules in the NIST 23 database raises concerns that machine learning models built using this database may be inaccurate for such molecules. Our database, despite its small size, may allow for improvements in such models. The database is available online in the Figshare repository: https://doi.org/10.6084/m9.figshare.30185032, accessed on 9 November 2025.

## Figures and Tables

**Table 1:** Compounds for which information on retention indices *I* is contained in the *mini*-N*_i_* database, and for at least one of the stationary phase types (5%-phenylpolydimethylsiloxane, designated as SSNP, or polyethyleneglycol, designated as SP) is not available in the NIST 23 database.

Name	*I_SSNP_*	*I_SP_*	Name	*I_SSNP_*	*I_SP_*
1,3,5-Triazine ^a^	678	1171	3-Amino-5-methylpyrazole ^c^	1165	2548
Formamide ^b^	694	1796	1-Methyl-2-imidazolemethanamine ^c^	1190	-
2-Methyl-1-pyrroline ^c^	758	1090	1,2,4,5-Tetramethylimidazole ^c^	1214	2033
Dimethylisoxazole ^c^	818	1280	1,3,5-Trimethyl-1H-pyrazol-4-amine ^c^	1216	2810
1-Ethylpyrazole ^a^	819	1279	4-Methyl-1,2,4-triazole ^c^	1218	2619
1,5-Dimethyl-1H-pyrazole ^c^	891	1403	3-Amino-5-methyl-4H-1,2,4-triazole ^c^	1251	3012
1-Ethyl-1H-1,2,4-triazole ^a^	914	1596	2,6-Diaminopyridine ^a^	1262	2588
1-Vinylimidazole ^c^	947	1685	3,5-Diisopropylpyrazole ^a^	1263	2025
2-Aminopyrimidine ^a^	975	1892	1-(3-Aminopropyl)imidazole ^c^	1346	-
1,2,5-Trimethylpyrrole ^a^	1001	1412	2-(5-Aminopyrazol-1-yl)ethanol ^c^	1349	2809
1,4-Dimethyl-1H-1,2,3-triazole ^c^	1034	1878	3,4-Diaminopyridine ^c^	1410	-
2-Aminopyrazine ^c^	1060	2151	3-(3,5-Dimethyl-1H-pyrazol-1-yl)-3-oxopropanenitrile ^c^	1414	-
3,5-Dimethyl-1H-1,2,4-triazole ^c^	1064	2262	1-Methylbenzimidazole ^a^	1442	2509
2,4-Dimethylimidazole ^c^	1070	2194	1-Methyl-1H-indazol-3-amine ^c^	1504	2675
1,3-Dimethylurea ^c^	1078	2276	5-Amino-4-cyanopyrazole ^c^	1526	3799
3-Ethyl-1H-1,2,4-triazole ^c^	1086	2336	4-Amino-3,5-dimethyl-1,2,4-triazole ^c^	1608	3548
(1-Methyl-1H-pyrazol-5-yl)methanol ^a^	1097	2185	5-Aminoindazole ^c^	1652	-
1-Methyl-1H-imidazole-5-carbaldehyde ^b^	1098	1999	4-(1H-Pyrazol-1-ylmethyl)benzonitrile ^c^	1733	2974
2-Amino-5-methylpyrazine ^c^	1131	2149			

^a^—The NIST 23 database contains data for non-polar stationary phases, but no data for polar stationary phases; ^b^—the NIST 23 database contains data for polar stationary phases, but no data for non-polar stationary phases; ^c^—the NIST 23 database does not contain data for either non-polar or polar stationary phases.

**Table 2:** Molecules for which there is a significant discrepancy in retention indices (non-polar stationary phases) between the NIST 23 database and our results; values are provided from our *mini*-N*_i_* database and the NIST 23 database; the range of values observed in a series of experiments is given in parentheses; in the case of NIST 23, values are given for the stationary phase types “semi-standard non-polar” (SSNP) and “standard non-polar” (i.e., polydimethylsiloxane); unless otherwise specified, NIST 23 provides only one retention index value for the corresponding stationary phase.

Name	*mini*-N*_i_*	NIST, SSNP	NIST, Polydimethylsiloxane
4-Methylimidazole	1030 (1029–1031)	1198 *	-
N,N-Dimethylethylenediamine	744 (741–746)	-	820
2-Aminopyrimidine	975 (973–977)	-	1139
2,4-Dimethylpyrrole	931 (929–932)	842	842
2,6-Diaminopyridine	1262 (1257–1266)	1377	-
1H-Indazole	1308 (1305–1312)	1260	1383

*—the NIST database contains two records containing the same value (from articles by the same authors).

**Table 3:** Molecules for which a difference in retention indices (polar stationary phase) of more than 100 units is observed between our results and the data given in the NIST 23 database; the range of values observed in a series of experiments (in the “*mini*-N*_i_*” column) is given in parentheses; the number of records in the NIST 23 database and the number of sources from which these values were obtained (in the “NIST” column) are given in parentheses; the years of initial publication of the values are also indicated.

Name	*mini*-N*_i_*	NIST	Years
1-Methylpyrrolidine	989 (983–1008)	861 (9, 3)	1971, 1973, 1991
4-Aminopyridine	2413 (2406–2428)	2299(6, 3)	1973, 1987, 1977
1,3,5-Trimethyl-1H-pyrazole	1451 (1446–1466)	2197 * (1, 1)	2011
1H-Benzotriazole	3217 (3212–3224)	2629 * (1, 1)	2005
Propazine	2771 (2766–2792)	2633 (3, 3)	1978, 1979, 1979

*—the value was obtained without the use of standard samples in the tentative analysis of a complex mixture.

## Data Availability

The data obtained in this work are available in in the Figshare repository: https://doi.org/10.6084/m9.figshare.30185032, accessed on 9 November 2025; detailed explanations are given in Section 3.1.
